# A selective deficit in imageable concepts: a window to the organization of the conceptual system

**DOI:** 10.3389/fnhum.2013.00226

**Published:** 2013-06-18

**Authors:** Aviah Gvion, Naama Friedmann

**Affiliations:** ^1^Communication Sciences and Disorders Department, Ono Academic CollegeKiryat Ono, Israel; ^2^Language and Brain Laboratory, Sagol School of Neuroscience and School of Education, Tel Aviv UniversityTel Aviv, Israel; ^3^Communication Disorders Department, Reuth Medical CenterTel Aviv, Israel

**Keywords:** semantic lexicon, conceptual system, abstract concepts, imageability, Hebrew, aphasia

## Abstract

Nissim, a 64 years old Hebrew-speaking man who sustained an ischemic infarct in the left occipital lobe, exhibited an intriguing pattern. He could hold a deep and fluent conversation about abstract and complex issues, such as the social risks in unemployment, but failed to retrieve imageable words such as *ball, spoon, carrot*, or *giraffe*. A detailed study of the words he could and could not retrieve, in tasks of picture naming, tactile naming, and naming to definition, indicated that whereas he was able to retrieve abstract words, he had severe difficulties when trying to retrieve imageable words. The same dissociation also applied for proper names—he could retrieve names of people who have no visual image attached to their representation (such as the son of the biblical Abraham), but could not name people who had a visual image (such as his own son, or Barack Obama). When he tried to produce imageable words, he mainly produced perseverations and empty speech, and some semantic paraphasias. He did not produce perseverations when he tried to retrieve abstract words. This suggests that perseverations may occur when the phonological production system produces a word without proper activation in the semantic lexicon. Nissim evinced a similar dissociation in comprehension—he could understand abstract words and sentences but failed to understand sentences with imageable words, and to match spoken imageable words to pictures or to semantically related imageable words. He was able to understand proverbs with imageable literal meaning but abstract figurative meaning. His comprehension was impaired also in tasks of semantic associations of pictures, pointing to a conceptual, rather than lexical source of the deficit. His visual perception as well as his phonological input and output lexicons and buffers (assessed by auditory lexical decision, word and sentence repetition, and writing to dictation) were intact, supporting a selective conceptual system impairment. He was able to retrieve gestures for objects and pictures he saw, indicating that his access to concepts often sufficed for the activation of the motoric information but did not suffice for access to the entry in the semantic lexicon. These results show that imageable concepts can be selectively impaired, and shed light on the organization of conceptual-semantic system.

## Introduction

When a neuropsychologist uses the term “imageability effect” we usually understand that imageable words are better than abstract ones. Indeed, most studies that report an effect of imageability on naming in aphasia present individuals who name imageable target words more accurately than abstract ones. This can already offer some insight into the organization of the conceptual-semantic system. In the current study we examined in detail the opposite effect: we report on Nissim, a 64 years old man who sustained a left occipital stroke, who had good naming and comprehension of abstract words and concepts and impaired access to words and concepts that have visual attributes. A line of tests that we report below shows that Nissim had a selective impairment in the conceptual system that did not allow him to fully process concepts that have visual properties. This in turn did not allow him to go from a concept that has visual attributes to the lexical item, or to access such concepts from the semantic lexicon. Such dissociation, in which words and concepts that have visual attributes are impaired, alongside good access to abstract words and concepts, can be informative about the organization of the conceptual-semantic system.

The more frequently witnessed dissociation is the one in which the performance on concrete words is better than that of abstract words. Such pattern was reported in various papers. For example, Nickels and Howard (1995) showed an imageability effect in naming in three aphasic patients, as well as at the group level for 15 individuals with aphasia. Whereas Nickels and Howard made a distinction between concreteness and imageability, they suggested (as did Franklin et al., 1995), that both imageability and concreteness might assist naming by richness of semantic representation (rather than by the accessibility to sensory experience or imageability *per se*). Franklin et al. (1995) reported on the case of DRB, who showed specific difficulty in the retrieval of abstract words. Additional discussions of the imageability effect and reports of aphasic patients who showed significant imageability effect, with better performance on high imageability words compared to low imageability words were reported by Franklin (1989); Nickels (1995); Tyler and Moss (1997); Bird et al. (2000); Luzzatti et al. (2002); Crepaldi et al. (2006, 2012), and others.

This effect was also found for semantic dementia (SD) and Alzheimer's disease (AD) patients. For example, Jefferies et al. (2009) examined synonym judgment of high and low imageability words (in fact, they were looking to see whether a reversed imageability effect characterizes semantic dementia). They tested 11 patients with semantic dementia and found only the common effect, with better comprehension of imageable words than abstract ones. Yi et al. (2007) studied naming to definition in semantic dementia and Alzheimer's disease, and found the same direction of dissociation: both naming to definition and comprehension from definition were worse on abstract nouns than on imageable ones.

This direction of dissociation, with imageable words being better than abstract ones is evinced not only in aphasia and semantic dementia, but also in healthy individuals in a wide range of tasks (Bourassa and Besner, 1994; Walker and Hulme, 1999; see Paivio, 1991 for review). This direction of dissociation is also seen in various neuropsychological conditions such as deep dyslexia (Coltheart, 1980), and also in recalling early items in serial recall tasks, as shown in the performance of patients who suffer from phonological short term memory limitation (Saffran and Martin, 1990). This advantage has been generally ascribed to the assumption that concrete words have richer semantic representations (Plaut and Shallice, 1991, 1993; Nickels and Howard, 1995), or that concrete words benefit from having visual features in addition to their semantic features (Paivio, 1991).

The opposite effect, which we report in the current study, with better performance on abstract words than on imageable words, is less frequently reported. However, some patients were reported to show this direction of effect. Warrington (1975) described AB, a patient who showed poor picture recognition and picture-word matching, who had particular difficulty in defining spoken (low frequency) concrete words, but was better at defining abstract words. Warrington (1981) reported on CAV, who showed the same effect in reading, with better reading of abstract words than of concrete words, and showed considerable difficulty in naming objects and pictures. Later on Warrington and Shallice (1984) described SBY, who defined correctly 94% of the (low frequency) abstract words given to him, but only 50% of the (low frequency) concrete words. Another very thoroughly-tested case that clearly demonstrates this dissociation was described by Marshall et al. (1996). They reported a man with semantic jargon aphasia, RG, who named and understood abstract words better than imageable ones, in a list of tests: naming to pictures and to definitions, word to picture matching, word association, and synonym judgment. A similar pattern of better performance on abstract words was also reported for a patient with semantic dementia by Breedin et al. (1994). Their patient, DM, showed abstract word advantage on a wide range of tasks including word definition and synonym judgment tasks, as well as in spontaneous speech. Similarly, FB, reported by Sirigu et al. (1991), produced better definitions of abstract than of concrete words, produced more items in a verbal fluency task of abstract compared to concrete words, and his spontaneous speech also showed better production of abstract words. Yi et al. (2007), who tested semantic dementia participants showed this pattern for verbs, but not for nouns. They showed a more severe impairment on motion verbs, which are more imageable, compared to cognitive verbs, which are related to psychological mental states. Thus, albeit less common, a selective impairment in abstract words or concepts is also attested.

Other dissociations that shed light on the organization of the conceptual-semantic system come from the extensive literature on category-specific impairments in naming and comprehension, beyond the concrete-abstract dissociation. These dissociations have been reported for broad categories such as living versus non-living concepts or words, and also for more specific semantic categories, such as fruits and vegetables, animals, musical instruments, tools, body-parts, clothes, and gemstones (see De Bleser, 2009, for review), and double dissociations were also reported. For example, Warrington and McCarthy (1983, 1987) reported a dissociation with inferior performance in the production and/or comprehension of non-living things compared to living things and Warrington and Shallice (1984) reported the opposite side of dissociation, with better performance on non-living than on living items. Warrington and Shallice (1984) suggested that this double dissociation can be explained by the different semantic features involved in the semantic representations for living and non-living items. According to their account, identification of living things (e.g., animals) relies more heavily on visual features, whereas the representation of artifacts critically hinges on their function. Along these lines of visual-functional distinctions, the difference in semantic features within the representation of different concepts can also account for further selective deficits. For example, body parts have very salient functional features, and hence pattern with non-living entities, whereas the distinction between gemstones depend on visual features, like living things (see De Bleser, 2009 for a review).

In the current study we explore, using a long line of tasks and modalities, the ability of a patient with abstract-imageable dissociation to name from pictures, objects, definitions, and from tactile presentation, to understand words and sentences, to provide a gesture for a picture or a definition, to make lexical decisions, to repeat and to write to dictation. This extensive assessment allowed us to point to the specific locus of impairment that gives rise to the pattern he shows, and from there to learn about the conceptual-semantic system, the way it encode concepts with visual attributes, the way it encodes motoric information, and to shed further light on the effect of various semantic features in the representation of concepts.

## Participant

Nissim was a 64 years old right handed man, a native speaker of Hebrew. He was referred to the clinic following an ischemic left sub-acute occipital infarct. Upon arrival at the hospital, he was diagnosed with severe aphasia and right hemiparesis, right and left arm apraxia (which improved by the time we tested him), finger agnosia, dyscalculia, and right hemianopsia. CT revealed, in addition to the occipital infarct, chronic lacunar left caudate and right thalamus infarcts. He had 12 years of education, worked before the stroke as a guard in a children's day care center, and had no premorbid language, reading, or writing disorders. He was diagnosed with severe naming and comprehension deficits according to the Hebrew version of the WAB (Kertesz, 1982; Hebrew version by Soroker, 1997). His spontaneous speech was fluent, with semantic jargon, severe word-finding difficulties (which we later found out occurred when he searched for an imageable word), and press of speech. He was unable to read words.

At the time of the assessment reported below, Nissim was 3 months post his stroke. In spontaneous speech, he could discuss complex issues using abstract words, but failed to retrieve even very frequent imageable words. For example, we heard him hold a detailed conversation about the social risks of unemployment, where he could develop profound ideas using abstract words. Yet he failed to convey even the simplest information regarding what he ate for breakfast, or retrieve the names of his wife and children. When he described to us his failure to convey messages and to name objects or pictures, he said “I have become a person that has no answers. I don't have my words.” In his attempts to describe the WAB picture of the picnic scene, he produced semantic jargon, perseverations, and empty speech: “*A person that is guarding himself or guarding someone else through language that is here that appears quite clear and he actually he reads.*”

## The phenomenon: a dissociation between abstract and imageable

To examine the extent of Nissim's difficulty and the difference between imageable and abstract words and concepts, to examine whether the difficulty existed in comprehension as well as in production, and to find out whether it affects the lexical-semantic level or the conceptual level, we ran a series of tests of picture naming, tactile naming, and naming to definition, of word and sentence comprehension and association tasks, and gesture production.

### Picture and object naming

We tested Nissim's naming from the visual modality using picture-naming and object naming tasks.

#### Method

The *picture-naming* test (SHEMESH, Biran and Friedmann, 2004) includes 100 pictures of objects of various semantic categories. Nissim saw the pictures, each presented on a separate card for an unlimited time and was asked to say the object's name. The object names are one to four syllables long, 3–10 phonemes, with ultimate and penultimate stress and with various first phonemes. The target word frequency as estimated by a Hebrew corpus encompassing 165,000,000 written words (Linzen, 2009) was 0.2–485 times per million words (M = 24, SD = 76). The average performance of adults aged 50–70 without a language deficit in this test is 96% correct.

The *object naming* task included 16 daily objects 1–3 syllables long, 3–10 phonemes, with ultimate and penultimate stress and with various first phonemes. The target word frequency (Linzen, 2009) was 1–64 times per million words (M = 18, SD = 23).

#### Results

Nissim could not name any of the pictures. Because of his extremely poor performance and the deep frustration he expressed, we stopped the test after 15 pictures (0/15). He also could not name any of the objects presented to him visually (0/16).

His responses were failed definition attempts, semantic jargon, and perseverations (see examples in Table 1. The examples throughout this article are translated from Hebrew). There were only two instances in which he produced definitions that were relatively good definitions of the objects he attempted to name, which reflected semantic knowledge of the object.

**Table 1:** **Examples of Nissim's picture and object naming**.

**EXAMPLES OF PICTURE NAMING**	
Bike	A specific time to stroll with it to play with it (correct gesture of hands holding the bicycle handlebars), a use that a person would like to do with it hours. Sets the time to do with it
Coat	(Makes the correct gesture). To put it on me. I can adjust it when I want to put it on me for a specific time that I set for the weather. Who will be cold and warm for me
Cookies	For eating (an appropriate gesture of eating). (exp: What is it made of?) Vegetable, something spicy that has to do with the fruit of nature, fruit of the tree
**EXAMPLES OF OBJECT NAMING:**	
Ball	This is something. (shows a gesture of catching a ball). Something accurate, swift, accurate. (shows a gesture of throwing a ball). Something accurate that can serve him
Knife	To put it something for. Or future, something related to the next stages. (a gesture of cutting with a knife). You need to hold it on this side (showing)
Screwdriver	It is a tool that is working a human being. There is here the side that holds (pointing to the handle). (a gesture of holding a screwdriver, without the relevant action)
Comb	A tongue/language when one wants to write (a gesture of hair-combing)

Interestingly, whereas he made no attempt to use other names for the objects, and hence made no paraphasias, he was able to produce some superordinate category names, and to use abstract words in the definition attempts. The fact that he used superordinate names can be explained by accounts according to which the basic level of concepts is the highest level that can be imagined. Namely, a superordinate is not related to a single perceptual image (Rosch et al., 1976; Violi, 2001; Feldman, 2006). Hence, the name of a superordinate category, which is not related to a visual image, is easier for Nissim to retrieve.

Importantly, and as demonstrated in Table 1, although he was not requested to do so, alongside his attempts at the naming task, Nissim provided 17 correct gestures to the pictures and objects he failed to name (see Sirigu et al., 1991, for a patient with severe object identification problems who was still able to demonstrate object manipulations, and Lhermitte and Beauvois (1973), for a patient who could not name objects from the visual modality but correctly mimed their use).

### Tactile naming

To further examine the naming deficit and to find out whether it is specific to naming from the visual modality, we tested Nissim's tactile naming.

#### Method

Seven daily-used objects: a cup, a spoon, a key, a toothbrush, reading glasses, a cap, and a TV remote control were given to Nissim for tactile naming. Each object was put in Nissim's left hand (he was allowed to touch the objects with both hands) while his eyes were closed. He was requested to touch the object and grope it for as long as he needed, and then to name it.

#### Results

Nissim named correctly only one of the seven objects (14%). He produced five perseverations, for example, instead of naming the cap he said: “It seems to me like a musical instrument, not music”; a similar response was given for a tooth brush: “It may be a heavy musical instrument… it is a musical instrument,” reading glasses: “It can only be a musical instrument,” and a remote control: “This is music… it can be a musical instrument.” In addition he attempted to produce definitions for the target items but he managed to produce a partially relevant paraphrase only for a cup: “That we drink in a specific holiday something that belongs to a hot Passover.” It is worth mentioning that this task immediately followed a task in which he was requested to list as many holidays as he could in 1 min and then to list as many musical instruments in 1 min, so this is where the perseverations came from.

We already had a clue that Nissim's difficulty was not limited to naming from the visual modality, as he had imageable word finding difficulties also in spontaneous speech. The tactile naming task further stresses this conclusion, as Nissim showed very poor naming from the tactile modality as well.

In marked contrast with his inability to name the objects, he provided appropriate gestures to each of the seven objects he held. (He provided these gestures although he was not requested to do so). Even given the relatively small number of items in this task, the difference between his naming (1/7) and gesturing (7/7) was significant (using a McNemar test), *p* = 0.03. This, and his good spontaneous gesturing in the picture naming task, suggest that the information he gains from the object is enough to access the correct concept and to activate the gestural information in the concept that would then activate the correct entry in the praxicon.

### Naming to definition: abstract and imageable concepts

So far Nissim's naming was found to be severely impaired in visual and tactile presentations. To evaluate Nissim's ability to produce abstract words and to compare abstract and imageable words, this experiment tested his naming to definition of high and low imageability concepts.

#### Method

The test included definitions for 120 target items, 70 low imageability target words and 50 high imageability target words. We read to Nissim a definition of a word (a noun or an adjective) and he was requested to orally produce the word. For example, definitions for high-imageability concepts included: *A tool used for cutting bread; What does the hen lay*?, and the definitions for the low-imageability words included *What is information that is whispered in the ear and is not for distribution?; Assets that are left by someone after he passed away.* The definitions of the high and low imageability target words were presented together, in random order. Most of the definitions provided for the target imageable words included an imageable word (40/50) and most of the definitions for the abstract words were abstract (66/70). The target high and the low imageability words did not differ with respect to frequency (Hebrew frequency database, Linzen, 2009; *p* = 0.26).

#### Results

Nissim named correctly 56/70 (80%) of the low imageability words, but only 24/50 (48%) of the high imageability words. His naming of the low imageability words was significantly better than his naming of the high imageability ones, χ^2^ = 13.44, *p* = 0.0002. For example, he easily named *inheritance, elections*, and *advertising*, while failing to name imageable words such as *carrot, necklace*, or *chocolate*.

Analysis of the naming errors reveals that for the high imageability target words he produced 13 semantic paraphasias, 9 perseverations, 3 attempted definitions, 2 correct gestures that indicated that he accessed the concept, 2 partially correct gestures, and one incorrect gesture. In addition there were 3 (10%) correct but delayed responses. He made no phonological errors.

Analysis of the errors Nissim made for the low imageability target words reveals that he produced 4 semantic paraphasias, 3 repetitions of words from the question, 2 “don't know” responses, and 5 consecutive responses in which he was requested to provide the opposite of a word and instead he explained the word the experimenter said. Here, too, he made no phonological errors.

Interestingly, he did not have perseverative responses when he tried to retrieve the abstract words, indicating that the perseverations are entering a void left by words that do not activate an entry in the semantic lexicon. Table 2 presents examples of responses that he produced for definitions of imageable and abstract concepts.^1^

**Table 2:** **Examples of Nissim's responses in the naming to definition task: high and low imageability words**.

**Definition provided**	**Nissim's response**
**HIGH IMAGEABLITY WORDS**	
What do we wear on our feet?	*A coat*
What is the color of a cucumber?	*White, black? Green*
An orange vegetable that rabbits eat	*A tangerine. I know what you mean, you mean a tall-statured vegetable*
A sweet made of cacao, with milk or bitter flavors	*Honey*
The desert animal	*It goes up in two phases, it has some phases*
The shape of the ball	*(Nissim drew a circle with his finger but made no verbal response)*
The color of snow	*Pink*
An animal with a long neck	*A snake, no, it raises its head*
A traditional Hanukkah toy (a dreidel)	*A giraffe?* (this item appeared immediately after the experimenter told Nissim the correct name of “an animal with a long neck”)
**LOW IMAGEABLITY WORDS**	
The eldest son in a family	*Firstborn*
Arrives to a visit in a foreign country	*Tourist*
Information that is whispered in the ear and is not meant for further distribution	*Secret*
An imaginary story that one sees during sleep	*Dream*
A person who has a lot of money	*Rich*
A situation in which a person does not eat or drink, such as in Yom Kippur	*Fasting*
A song that represents a country	*Hymn*

Another analysis we have done related to the effect of operativity on Nissim's naming of imageable words. Concepts are defined as *operative* if they can be readily grasped, manipulated, and operated upon. Whereas some studies reported that operativity played a crucial role in participants' performance (Gardner, 1973; Howard et al., 1995; Nickels and Howard, 1995), Nissim showed no such effect (χ^2^ = 0.64, *p* = 0.42): he had 10/18 correct responses on operative concepts (which included mainly tools and kitchenware), and 14/32 correct on non-operative imageable concepts (such as the sun, or a giraffe).

### Naming high and low imageability proper names: naming to definition

The naming to definition test revealed a clear imageability effect in Nissim's naming, with better naming of low imageability words. The next test examined the imageability effect within one category: proper names. All the target words in this task were proper names, but some of the proper names were of people that are closely tied to a visual image, and others were names of people without a visual image (figures from the bible, for example). Some of the names in the two categories were the same name, which appeared once in the abstract condition (*Moshe Rabenu*, Moses) and once in the visual-image condition (*Moshe Dayan*).

#### Method

We orally presented to Nissim 31 descriptions of people, and he was asked to retrieve a name for each description. All the names were names of familiar people: well known politicians, actors, football players, singers, figures from the bible and Nissim's family members. Of the target proper names, 17 were people that are well known by their image, because they appeared in electronic and written media, or known specifically to Nissim because they are his family members. The 14 other proper names were names that are very familiar but are not connected to a visual image. The list of famous “imageable” people consisted of political leaders (such as the first Israeli prime minister, David Ben-Gurion), Nissim's family members (wife and children), and famous football players^2^ (Maradona, Pelé). The list of the familiar “non-imageable” people consisted of biblical figures (such as Abraham and King David) and famous Israeli early twentieth century poets. The descriptions did not include any visual properties of the person described.

#### Results

Nissim named the low imageability names (10/14, 71%) significantly better than he named the high imageability ones (6/17, 35%), χ^2^ = 4.01, *p* = 0.045. Whereas he could name Moshe, the biblical Moses who does not have a visual image related to him (at least not in Judaism, where religious figures are rarely depicted), he failed to name Moshe Dayan, a well known Defense Minister and Foreign Minister of Israel, who was very tightly connected with a well-defined visual image, which included an eye patch. Whereas he could name Isaac, the son of biblical Abraham, he could not retrieve the names of his own sons.

Nissim's incorrect responses for the high imageability proper names included 4 definitions, two of which did not convey any accurate information, 4 “don't know” responses, 3 semantic paraphasias, and one perseveration. As for the low imageability proper names there were 2 “don't know” responses, 2 definitions, and one correct but delayed response (see Table 3 for examples).

**Table 3:** **Examples of Nissim's responses in the naming to definition task: high and low imageability proper names**.

**DEFINITIONS OF HI PROPER NAMES**	**RESPONSE**
The current defense minister (Ehud Barak)	*A man a bit younger than me (demonstrates with his hand his height) very famous, was the father of the daughter*…. not Ez….
Nissim's elder son (Yoram)	*He lives at my house every day*
Nissim's second son (Avi)	*I have three, one was my eldest son, i.. my daughter… he was in a high rank… Ran?*
The current prime minister (Binyamin Netanyahu)	*Begin* (a former prime minister)… *Peres* (the current president)… *Eshkol* (a former prime minister)
The defense minister during Yom Kippur war, who was also a chief of staff and a minister of foreign affairs, an amateur archaeologist, and a women lover. (Moshe Dayan)	*David?*
**DEFINITIONS OF LI PROPER NAMES**	**RESPONSE**
The female poet who wrote the song about the lake of Galilee, who died of Tuberculosis. (Rachel)	*Rachel*
The Hebrew leader of the Egypt Exodus (Moses)	*Moses… Pharaoh… Moses*

### Picture naming: proper names

We also tested Nissim's production of proper names using a picture naming task. Naturally, in this test all target people were easily identifiable by their picture, and hence, imageable.

#### Method

Nine color pictures of famous people, 7 politicians (for example *Bill Clinton*) and 2 famous singers (*Elvis Presley*) were introduced for naming.

#### Results

Nissim could not name any of the pictures (0/9 correct). He produced only one name, which was incorrect (naming the picture of Elvis Presley “*Shimon Peres*,” the Israeli president). For each of the pictures he attempted to provide semantic information about the person in the picture, but none of these definitions was accurate. For two of the pictures he produced some relevant information. For example, when he saw the picture of Elvis Presley he said: “Peri.. Peri… Shimon Peres… he was the leader number one… he will last for a long time on top of the calibers of the type of music.”

### Comprehension of imageable words: word-picture matching

The previous tests clearly indicated Nissim's severe deficit in the production of imageable words. We now assessed whether the same deficit applies to his word comprehension.

#### Method

Auditory comprehension was tested using a spoken word to object/picture matching task, taken from the Hebrew version of the WAB (Kertesz, 1982; Hebrew version by Soroker, 1997). This subtest includes five 6-item sets (real objects, pictures of the same objects, letters, numbers, and colors). Nissim heard a word that matched one of the six pictures/objects in the set, and was requested to point to the matching picture/object.

#### Results

Nissim performed only 5/30 correct on this test, where the guessing pattern distributes around 5/30. Namely, he showed a guessing pattern. His performance in each category was at or just below chance level: 3/6 correct in real objects, 1/6 correct in the pictures of the same objects, 1/6 correct in the color category, and 0/6 in the letters and numbers sets.

### Comprehension of high and low imageability words: word association task

To compare between Nissim's comprehension of high and low imageablity words, we administered a word association task. Nissim heard triads of words: a target word and two other words, and was requested to choose which of the two words is semantically related to the target word. For example: “What relates to shoes, hands or feet?” He was asked to say the word (feet) or to say “The first word/the second word.”

#### Method

The test used the *MA KASHUR* word association task (Biran and Friedmann, 2007b), to which we added six triads. In total, the task included 39 triads of words, 27 triads of high imageability words, and 12 triads of abstract words. For example: imageable triads: shoes—hands/feet; cow—milk/coke. Abstract triads: honesty—truth/lie; Education—enlightenment/primitiveness. The target high and low imageability words did not differ in frequency [*t*_(37)_ = 0.61, *p* = 0.55], based on Linzen (2009) Hebrew frequency database.

#### Results

Similarly to the high/low imageability dissociation in production, in this task too, Nissim performed significantly better on the low-imageability words, with 12/12 (100%) items correct, than in the high imageability sets, in which he was correct only on 20/27 (74%) of the items, χ^2^ = 3.79, *p* = 0.05. For example, he incorrectly chose the word *bag* (rather than *pillow*) as related to *bed*, and chose *a door* (instead of *window*) as related to *curtain*. However he correctly associated the word *crime* to *prison* and not to *award* and *time* to *seconds* and not to *kilos.*

### Comprehension of high imageability words: a surprising dissociation between objects and body parts

#### Comprehension of high imageability words within sentences

Another way to assess Nissim's comprehension of words was to test high imageability words within sentences. This was evaluated through the analysis of his performance in the Sequential Commands subtest of the WAB.

#### Method

The 11 commands in this subtest include 20 imageable nouns: 18 names of objects in the room (“Point to the chair”) and 2 body parts (“Raise your hand”). We examined for each of the nouns whether Nissim was able to identify it (by manipulating the relevant object) or not.

#### Results

Nissim, again, showed very poor comprehension of the objects, and did not perform correctly any of the 9 commands that included an object. However, surprisingly, he performed well on the two commands that involved his body parts—his hand and his eyes.

#### Comprehension of names of body parts

To further explore this relatively preserved comprehension of the names of his body parts, we used the body parts and the right/left body parts subtests of the auditory comprehension WAB test, in which Nissim was requested to point to 9 of his body parts when he heard their names (point to your ear, nose, eye, stomach, neck, chin, nails, palm of the hand, arm), and then 7 body parts for which we also specified the side (your right ear, right shoulder, left knee, left ankle, right hip, left elbow, right cheek).

#### Results

Whereas, as reported in the Word-Picture Matching Section, Nissim performed at chance level with objects and pictures in the auditory comprehension task, he performed relatively well when the task required him to point to his own body parts (15/16 correct), and his performance on the body parts was significantly better than his performance on the objects and pictures, χ^2^ = 30.73, *p* < 0.0001.^3^

A possible explanation for Nissim's better performance with pointing to his body parts is that his body parts are encoded proprioceptively, and not visually. Sadly, we did not test his comprehension of pictures of body parts to examine this hypothesis.

### Comprehension of high and low imageability sentences: sentence verification task

To evaluate Nissim's comprehension beyond the word level, we tested his sentence comprehension using the sentence verification task of the WAB.

#### Method

The task involved 8 sentences that include high imageability words, such as: Is the door closed? And 9 items that included only low imageability words, such as: Does March precede June? Nissim heard each sentence and answered the question.

#### Results

As in the single word level, in the sentence level, too, Nissim showed a clear dissociation between sentences with high and low imageability words. Whereas he performed at ceiling (9/9 correct) on the low imageability sentences, he performed poorly and at chance level on the high imageability ones (5/8) with a significant difference between the conditions, χ^2^ = 4.1, *p* = 0.04.

### Comprehension of complex abstract concepts: interpretation of proverbs

The single word comprehension tests indicated that Nissim is not only impaired in the production of imageable words, he also struggles with the comprehension of imageable words. The sentence comprehension task showed that he also fails to understand simple sentences that involve imageable words, whereas he comprehends simple abstract sentences well.

In the next experiment we went beyond the single word level and the simple sentence level, and assessed Nissim's comprehension of proverbs. We selected proverbs for which the literal meaning is highly imageable, both because they include imageable words and because the literal meaning of the phrase or sentence as a whole is imageable. The meaning of the proverb, i.e., its figurative meaning was, however, abstract. This allowed us to test whether he could reach the abstract interpretation even when the literal meaning is highly imageable. This would enable us to examine whether the inability of Nissim, based on the findings so far, to extract the imageable, literal reading of the proverb blocks him from extracting the abstract figurative meaning of the proverb. Beyond telling us something about Nissim's impairment, it would also assist in a long-standing discussion in the literature of proverb comprehension: do we have to pass through the literal meaning to access the figurative one?

#### Method

The task included 13 proverbs, which were auditorily presented one by one. We selected proverbs for which the literal meaning was highly visually imageable, but their figurative proverb interpretation was abstract. After hearing each proverb Nissim was requested to explain the proverb's meaning in his own words.

#### Results

Nissim correctly described the meaning of 10/13 proverbs (77%, see Table 4 for examples). In four of these proverbs he correctly explained the proverb after it was put in a sentence. Interestingly, even in the three proverbs he did not explain correctly, he never provided an interpretation that was based on the literal meaning of the proverbs. Rather, in these cases he produced vague general responses for which we could not be sure that he interpreted the proverb correctly.

**Table 4:** **Examples of Nissim's proverb explanation**.

**Proverb**	**Nissim's explanation**
A broken reed	A person that is trusted to be about to help and save the situation but it turns out that he is a broken reed. It is a belief in something. He let down, he does less than he could do. He was relied upon more than he can help, economically or physically. He was trusted more… “What an asset there is here,” but there is nothing
(Danni entered) Like a stormy wind	He went inside very quickly, went quicker than he had planned, went in a way of ecstasy, nerves, stressed. More unrelaxed than he should have been
Jumped higher than his navel	Did more than he thought he needs to, jumped above his ability
Stood on his hind legs	He insisted
(Yossi is Moshe's) right hand	It means that he will stand by him physically, safety-wise, emotionally. He is his right hand, he stands by him

***Interim discussion: theoretical implications of proverb comprehension.*** These results shed light on a discussion regarding the process of access to the figurative meaning of proverbs (Temple and Honeck, 1999; Keysar et al., 2000). At this point in the study we can already safely conclude that Nissim cannot access visual aspects of concepts. His good performance in the comprehension of proverbs suggests that it is not necessary to go through the literal meaning of the proverb, which in this case was rich in visual features, in order to access the figurative meaning. In a way, his extreme difficulty in accessing words that had visual attributes, even if it seemed that he had enough information to access them without the visual attributes, suggests that the visual attributes block his word access. Thus, his good comprehension of proverbs might suggest something stronger than that the figurative comprehension meaning can be accessed without accessing the literal one. It might suggest that some inhibition on the literal meaning is active in normal interpretation of proverbs, which allowed Nissim to access their meanings correctly.

### Describing physical attributes of famous people

The tests up to now indicate that Nissim has tremendous difficulty in accessing words and proper names for concepts that include visual attributes. He also found it difficult to fully understand words that include visual attributes. We next tested his ability to describe visual attributes of people, when given their names.

#### Method

We said the names of 7 famous political leaders, and Nissim was requested to describe how they look. All of these people have a typical visual feature. For example *Theodor Herzl* (father of modern political Zionism) had a full, medium-long black beard; *Moshe Dayan*, a past Israeli Defense Minister and Foreign Minister, was well-known for his eye patch.

#### Results

Whereas none of Nissim's verbal descriptions were accurate, four gestures, for two of the people, conveyed relevant information about the person he was describing. This suggests that the gestures have better access not only to motor-gestural information about concepts but also to some visual information. For example, for Moshe Dayan, he showed, with his hand, an eye patch on his eye, but said “in one leg he had no hair in the right leg. A person without hair.” and showed an eye patch again with his hand. In other cases he knew what the characteristic dimension of the person was, but could not decide where the person was on this dimension (in a way similar to his ability to name the superordinate categories of objects he could not name). For example, when asked to describe Napoleon, he could say that his dominant visual feature was his height, but then continued to say “wasn't he the tallest person in the world?”. When asked to describe Barack Obama, he said: “Hair? color? In the face maybe? A color a bit darker than usual?”

This difficulty may be attributable either to difficulty in fully accessing the concept from the semantic lexicon, to a difficulty in the visual features in the conceptual system.

## What is the locus of the deficit?

**Figure 1:**
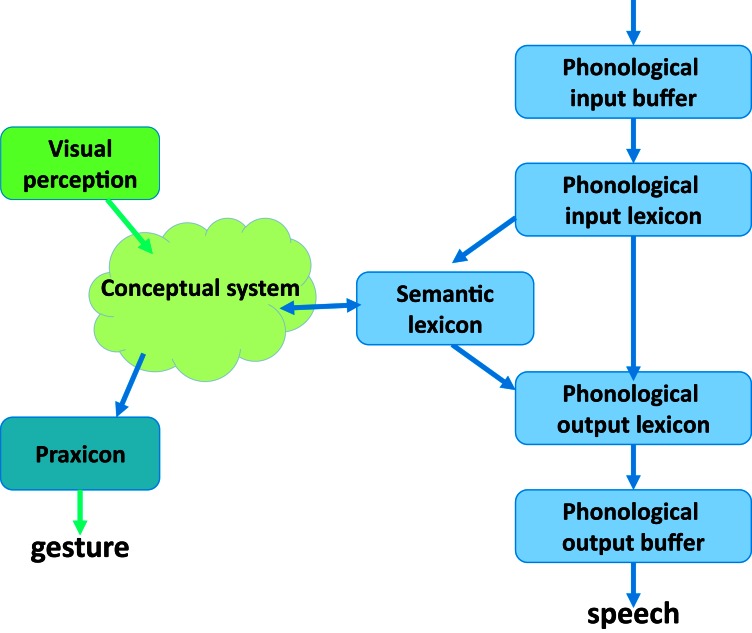
**Lexical processing model**.

The next step is to try and further focus on the locus of deficit that gives rise to Nissim's selective pattern of impairment. We assume a multi-stage model of lexical processing, schematically shown in Figure 1. For production, the first stage is the conceptual stage, in which a non-verbal message is created (possibly after the identification of an object, in case of object naming), followed by access to the appropriate entry in the semantic lexicon that includes words organized semantically, and then a phonological lexicon that holds phonological information about the word, and a phonological output buffer (Butterworth, 1989; Levelt et al., 1999; Nickels, 2001, 2002; Friedmann et al., 2013). For the input route we assume a phonological input buffer after the first auditory stages, which is followed by a phonological input lexicon, and then the semantic lexicon and the conceptual system, which are shared with the output process.

At this point we know that Nissim had difficulties in the production of imageable words from visual and tactile presentation of objects, from definitions, and in spontaneous speech, as well as in the comprehension of imageable words. We will now examine whether the visual processing is impaired and whether it can be the source of his difficulty, and then move to examine the various lexical and conceptual stages of word processing and find the component that is responsible for his pattern of impairment.

### Visual agnosia? a test of visual perception

We already saw in the previous experiments that Nissim's impairment could not be ascribed solely to a deficit in visual processing, as he made errors in tactile naming, naming to definition, and spontaneous speech, which do not involve visual perception.

To further examine whether the failure to name from the visual modality may be ascribed to visual agnosia, we tested Nissim's visual perception.

#### Method

We administered the visual perception subtests in the *LOTCA* (Loewenstein Occupational Therapy Cognitive Assessment, Katz et al., 1989). This test examines visual discrimination, visual memory, visual-spatial relationships, visual form constancy, visual sequential memory, visual figure-ground, and visual closure.

#### Results

Nissim performed flawlessly on the *LOTCA*'s visual perception subtests, reaching the maximum score in each of the subtests. This good performance indicates that his impaired performance in the picture and object naming tests and in the spoken word to object/picture matching task cannot be ascribed to a visual perception deficit.

### Phonological input and output abilities: auditory lexical decision, word and sentence repetition, and writing to dictation

Given Nissim's poor word production and comprehension, we evaluated his input and output phonological lexicons and buffers. We did so using an auditory lexical decision task, word, word sequence, and sentence repetition tasks, and writing to dictation.

Nissim's auditory lexical decision, assessed using *PALPA* 5/1 (Kay et al., 1992; Hebrew version by Gil and Edelstein, 1999) was very good. He performed 100% correct in this task (46/46), indicating that his phonological input lexicon was spared, and so was the path to it from auditory presentation (auditory perception, phonological input buffer).

His repetition of single words and sentences in the WAB (six 1–3 syllable high-imageablity single words and nine phrases and sentences of 2–10 words) was also good (with a final score of 92/100). He also repeated well 6 sequences of two unrelated 2-syllable words and eight sequences of three 2-syllable words (FriGvi, Friedmann and Gvion, 2002; Gvion and Friedmann, 2012). This supports the previous conclusion that his input phonological buffer and lexicon are spared, and further indicates that his phonological output buffer was spared, and given that he repeated well sentences that included up to 10 words, his phonological output lexicon is probably also spared, supporting the repetition of this large amount of phonemes.

Importantly, his repetition of imageable words, as single words and within sentences, was spared, indicating that he can retrieve imageable words from the phonological output lexicon, and the deficit in naming of imageable words lies in an earlier stage of the process, in the conceptual of lexical-semantic stages. This finding can also shed light on the repetition process, showing that it can proceed without going through the conceptual system.

The effects on his naming also pointed in the same direction of intact phonological stages: whereas he showed a very strong imageability effect as we saw before, he showed no length effect (with 20%, 16%, 30%, 33%, 25% correct performance in 2, 3, 4, 5, 6 and more letter words, respectively), excluding the phonological output buffer as the source of his impairment. He also showed no frequency effect (χ^2^ = 0.02, *p* = 0.09), ruling out a phonological output lexicon impairment.

A further instantiation of Nissim's preserved input phonological lexicon is his good writing to dictation. We dictated to Nissim 52 words (from the TILTAN writing screening task, Friedmann et al., 2007). He showed some errors that are mainly attributable to incorrect allograph selection (15 errors), 6 letter omission/migration that are typical for graphemic output buffer deficits (Yachini and Friedmann, 2009), and 4 sublexical writing errors (surface dysgraphia-like errors). However, importantly, he did not make errors in writing that even slightly resembled his difficulties in oral naming. There were 33 imageable words in this tasks, and he wrote all of them. This indicates, again, that his phonological input buffer and lexicon are intact.^4^ Given the pattern of Nissim's poor access to the semantic lexicon of imageable words, this indicates that he does not go through the conceptual-semantic system in writing to dictation.

### A conceptual deficit? picture association task

Given Nissim's relatively good performance in the visual tasks, his impaired imageable word production and comprehension, and his spared phonological input and output buffers and lexicons, two possible loci in the model remain that could give rise to his pattern of deficit: the conceptual system or the semantic lexicon (or the connection between them). To explore this question and decide between these two possibilities, we examined Nissim's conceptual abilities in a picture task that did not involve words and hence, did not involve the semantic lexicon, only the conceptual system.

#### Method

Nissim's conceptual ability was tested using a picture association task (*MA KASHUR*, Biran and Friedmann, 2007a). This task includes 35 triads of colored pictures. Each triad included a top picture, and a pair of pictures below it, from which he was requested to choose the picture that was semantically related to the top picture. For example, he saw a picture of a glove, and had to choose between a hand and a foot; or a picture of bread, and underneath it a knife and scissors. The foil in all triads was related visually or semantically to the other picture but not to the top one.

#### Results

Nissim performed 23/35 (71%) correct, a performance that is not significantly different from chance, using the binomial distribution. Furthermore, even when he made a correct choice he frequently hesitated and commented that he does not know or is unsure that this is indeed the correct picture. This indicates a deficit in Nissim's ability to associate two pictures on the basis of conceptual knowledge. Namely, even when no words are involved, the difficulty is already present, indicating that that the deficit lies in the early stage of the concept itself, prior to the access to its verbal representation in the semantic lexicon.

The comparison of his performance in this test to the parallel word association task (of imageable items) reported earlier indicated a similar and poor performance (71% vs. 74%, χ^2^ = 0.50, *p* = 0.48) in the picture and word tasks. This further points to the conceptual system as the source for Nissim's deficit. Had the deficit been located at the semantic lexicon or in the access to it from the conceptual system, we would have expected Nissim's performance on the picture association task to be good, and better than in the word association task. (Individuals with a semantic lexicon impairment perform well on this test that involves only pictures, and fail on the parallel word association test, see for example Biran and Friedmann, 2012).

Thus, all these considerations point to a selective deficit in the conceptual system that affects imageable concepts, and specifically the visual attributes within imageable concepts.

## Discussion

This case study showed a clear pattern of dissociation between abstract and imageable concepts. The participant was unable to retrieve words for imageable concepts in a variety of tasks: picture naming, object naming, naming to definition and tactile naming. He also failed in understanding imageable words. His deficit was also evident in a test in which he was requested to find semantic associations between pictures. When trying to retrieve imageable words, he made attempts at definitions, which were often incorrect, some semantic paraphasias, and many perseverations. In contrast, his production and comprehension of abstract words were relatively good, and did not give rise to perseverations. His ability to perform gestures for pictures and objects was much better preserved than his retrieval of the names of the same objects. Additional tests indicated that his visual perception, as well as his phonological input and output lexicons and phonological input and output buffers were intact.

This pattern of impairment and sparing indicates that Nissim's deficit lies in a selective impairment in the conceptual system. The picture that emerges from his performance suggests that concepts in the conceptual system are multi-faceted. A concept of an object, for example, includes its visual attributes, semantic features, functional features, and motoric-gestural information (see for example Shallice, 1988 and his discussion there of Allport, 1985). We suggest that Nissim's impairment lies in the visual properties within each concept. As a result, Nissim is often able to roughly access the relevant concept from a picture, from seeing or touching an object, or from a definition, in a way that provides him with enough information to access the motor features of the object, and retrieve the relevant gesture from the praxicon, but the information contained in the concept is not enough for him to access the entry in the semantic lexicon, or, in the other direction, to access the relevant concept from the semantic lexicon. Notice that we do not talk here about “richness of concepts,” as has been, for example, suggested by Franklin et al. (1995) and Nickels and Howard (1995) for the superior access to imageable concepts for some patients. Had it been simply a matter of richness of concepts, we would expect Nissim to be able to access the names, for example, of his sons, for whom he no doubt has a rich semantic representation. Instead, we suggest that it is the impairment of the visual features in the concepts that hinder the access to the name in the semantic lexicon.

One can think about Nissim's inability to access the semantic lexicon from the conceptual system as a case of insufficient information, or as a case of blocking, in which the inability to access the visual information is blocking further lexical access. The absence of phonological errors and his good performance in auditory lexical decision, in word and sentence repetition, and in writing to dictation show that his phonological lexicons and buffers are intact, and point to the conceptual system and, specifically to the visual attributes within the concepts as the source of his deficit (See Figure 1). For concepts without visual attributes, the processing of the concept in the conceptual system and the access from it to the semantic lexicon are more successful, because the information in the concept suffices to access the lexical entry (under the no-sufficient-visual-information explanation), or because no blocking occurs, as access to visual information is not required. The results also suggest that the visual attributes of the concept are not required for retrieving the appropriate gesture from the output praxicon, where the physical attributes of familiar gestures, such as their kinetic parameters, are stored and activated (Gonzalez Rothi et al., 1991; Heilman and Gonzalez Rothi, 1993). This would explain the good pantomime that Nissim was able to present when he failed to retrieve a name of an object.

This explanation follows in the footsteps of several researchers who accounted for various selective naming impairments in terms of the semantic features in the representation of concepts, such as Warrington and Shallice (1984); Franklin et al. (1995) and Nickels and Howard (1995). We do not assume (or exclude) here separate modality-specific semantic systems (see discussions for and against modality-specific semantic systems in Shallice, 1987, 1988; Caramazza et al., 1990; Hillis et al., 1990) but rather discuss the features internal to the each concept within the conceptual system.

The dissociation between concepts with and without visual attributes applied both for objects and for proper names. Just like in other nouns and adjectives, Nissim was able to retrieve names of people who are not related to a visual image, but failed to retrieve names of people who are tied to a visual image. The case of proper names is especially interesting because, unlike other nouns, the exact same proper name can be related to a person whose visual image is part of his concept (like Abraham Lincoln), and to a person with no visual image (the biblical Abraham). The finding that Nissim showed the same abstract-imageable dissociation in proper names suggests that the conceptual storage of person information is similar to the one described above: some people are stored with visual attributes, in which case Nissim fails to retrieve their names or appropriate information about them, whereas others, biblical figures for example, are stored without visual properties, and hence are accessed more easily by Nissim.

Another result that has interesting theoretical bearing is Nissim's good comprehension of abstract proverbs for which the literal meaning is highly imageable. Researchers of the process of access to proverbs' figurative meaning debate as to whether access to the figurative meaning is obligatorily preceded by a stage of access to the literal meaning of the concept (Temple and Honeck, 1999; Keysar et al., 2000). Nissim's good comprehension of proverbs with highly imageable literal meanings is thus very informative in this debate. Given that Nissim cannot access imageable concepts from words, his good comprehension of the figurative meaning of proverbs suggests that he did not go through a phase of accessing the literal meaning of the proverbs. More generally, this may suggest that it is not necessary to go through the literal meaning in order to access the figurative meaning of proverbs. According to a blocking account of his performance, i.e., that the existence of a visual image in the concept actually blocks further processing, the results might even suggest that the figurative meaning of proverbs involves inhibition of the literal meaning, which explains how come Nissim was not blocked in accessing the figurative meaning of the proverbs.

Another interesting point relates to the source of perseverations. Whereas Nissim's speech was replete with perseverations when he tried to retrieve imageable words, he had no or almost no perseverations when the target had no visual attributes. A similar tendency for perseverations when the target words are imageable seems to characterize also the error examples Warrington and Shallice (1984, p. 842) provided from SBY attempts to define highly imageable words. This suggests that the origin of the perseverations can be the attempt to produce output when no entry in the semantic lexicon is activated. In this case, the semantic lexicon does not pass on information to the phonological output lexicon so a word that is left activated from previous production in the phonological output lexicon is used instead.

Finally, previous studies described *optic aphasia*, a deficit in which the patient cannot name visually presented objects, but is able to identify them correctly by sight and to name them when they are presented in another sensory modality (Lhermitte and Beauvois, 1973; Beauvois, 1982; Davidoff and de Bleser, 1993; Luzzatti et al., 1998; Luzzatti, 2003). Whereas, similarly to cases of optic aphasia, Nissim was unable to name visually presented objects, his impairment clearly differed from optic aphasia. Beauvois (1982) clearly defines optic aphasia, and determines that the naming impairment in optic aphasia is specific to the visual modality. According to her the term “optic aphasia” is appropriate only for cases of normal language abilities, without anomia in speech production, and with normal spontaneous speech (as was the case with the patient reported in Lhermitte and Beauvois, 1973, for example). Because Nissim showed the same difficulty in concrete words in spontaneous speech, as well as in naming to definition and naming of tactilely presented objects, the diagnosis of optic aphasia does not seem to apply to him.^5^

### Conflict of interest statement

The authors declare that the research was conducted in the absence of any commercial or financial relationships that could be construed as a potential conflict of interest.

## Footnotes

^1^Interestingly, when one talks about “imageability” it is unclear which sensory modalities enter this definition. Most of our investigation involved the visual modality. We only touched this point by asking Nissim to name to definition two concepts with auditory attributes (what appears after the lightning? Which sound does a dog make?) and three tastes (what is the taste of sugar/lemon/Bamba). He succeeded in both auditorily-related names, and in 2 of the 3 tastes. (For the taste of Bamba, a Israeli peanut-based snack, he said “Blue, sweet, no… reddish mint?”, possibly failing because the target word given to him was highly visually imageable). These are too few items to make a solid conclusion but they may be carefully taken to suggest that Nissim's deficit was specifically related to visual attributes, rather than to general sensory ones.

^2^Nissim's primary hobby was sports and he was particularly very knowledgeable about football players history.

^3^He did, however, make three left/right errors when he was required to point to a body part on a specific side of his body. Although right/left disorientation is a primary symptom of Gerstmann's syndrome (Wilkins and Brody, 1971; Mayer et al., 1999; Roux et al., 2003), and he also showed dysgraphia and finger agnosia, which are characteristic of this syndrome, we do not think he should be diagnosed with this syndrome: a. His finger agnosia, or rather, his inability to name fingers, was probably part of his general inability to name concrete objects. b. Pure cases of Gerstmann's syndrome do not show any difficulty in language other than the inability to name fingers and numbers (Roeltgen et al., 1983; Cipolotti et al., 1991; Mayer et al., 1999; Rusconi et al., 2010), which is clearly not the case for Nissim, who had vast problems in spontaneous speech and in naming of all kinds of objects in various tasks. c. Gerstmann's syndrome is typically a result of a parietal damage (Rusconi et al., 2010), but Nissim had an occipital damage.

^4^As for the implication of his performance in this task about the status of the phonological output lexicon—this depends on the path one assumes for spelling to dictation: if the input arrives to the orthographic output lexicon directly from the phonological input lexicon, then this finding has no bearing as to the phonological output lexicon. If, however, the information goes from the phonological input lexicon via the phonological output lexicon to the orthographic output lexicon, then his writing performance also supports the conclusion that his phonological output lexicon is intact.

^5^Beauvois et al. (1978) and Beauvois (1982) discuss the possible effect of a visualization strategy on performance in tasks that do not straightforwardly involve the visual modality, like tactile naming. Whereas one may suggest that such strategy was involved in Nissim's difficulty in naming proper names to definition and in tactile naming, we believe that it is less plausible in accounting for his severe concrete word deficit in spontaneous speech.
